# Ultralight Magnetic and Dielectric Aerogels Achieved by Metal–Organic Framework Initiated Gelation of Graphene Oxide for Enhanced Microwave Absorption

**DOI:** 10.1007/s40820-022-00851-3

**Published:** 2022-04-19

**Authors:** Xiaogu Huang, Jiawen Wei, Yunke Zhang, BinBin Qian, Qi Jia, Jun Liu, Xiaojia Zhao, Gaofeng Shao

**Affiliations:** 1 Institute of Advanced Materials and Flexible Electronics (IAMFE), School of Chemistry and Materials Science, Nanjing University of Information Science and Technology, Nanjing, 210044 People’s Republic of China; 2 Department of Chemical and Biological Engineering, Monash University, Victoria, 3800 Australia; 3 College of Field Engineering, Army Engineering University of PLA, Nanjing, 210007 People’s Republic of China; 4 Hebei Key Laboratory of Inorganic Nano-Materials, College of Chemistry and Materials Science, Hebei Normal University, Shijiazhuang, 050024 People’s Republic of China; 5 School of Chemistry and Environmental Engineering, Yancheng Teachers University, Yancheng, 224002 People’s Republic of China

**Keywords:** Magnetic and dielectric aerogels, Metal–organic frameworks, Gelation mechanism, Microwave absorption, Radar cross-sectional simulation

## Abstract

**Highlights:**

- Metal–organic frameworks (MOFs) are used to directly initiate the gelation of graphene oxide (GO), producing MOF/rGO aerogels.
- The ultralight magnetic and dielectric aerogels show remarkable microwave absorption performance with ultralow filling contents.

**Abstract:**

The development of a convenient methodology for synthesizing the hierarchically porous aerogels comprising metal–organic frameworks (MOFs) and graphene oxide (GO) building blocks that exhibit an ultralow density and uniformly distributed MOFs on GO sheets is important for various applications. Herein, we report a facile route for synthesizing MOF/reduced GO (rGO) aerogels based on the gelation of GO, which is directly initiated using MOF crystals. Free metal ions exposed on the surface of MIL-88A nanorods act as linkers that bind GO nanosheets to a three-dimensional porous network via metal–oxygen covalent or electrostatic interactions. The MOF/rGO-derived magnetic and dielectric aerogels Fe_3_O_4_@C/rGO and Ni-doped Fe_3_O_4_@C/rGO show notable microwave absorption (MA) performance, simultaneously achieving strong absorption and broad bandwidth at low thickness of 2.5 (− 58.1 dB and 6.48 GHz) and 2.8 mm (− 46.2 dB and 7.92 GHz) with ultralow filling contents of 0.7 and 0.6 wt%, respectively. The microwave attenuation ability of the prepared aerogels is further confirmed via a radar cross-sectional simulation, which is attributed to the synergistic effects of their hierarchically porous structures and heterointerface engineering. This work provides an effective pathway for fabricating hierarchically porous MOF/rGO hybrid aerogels and offers magnetic and dielectric aerogels for ultralight MA.
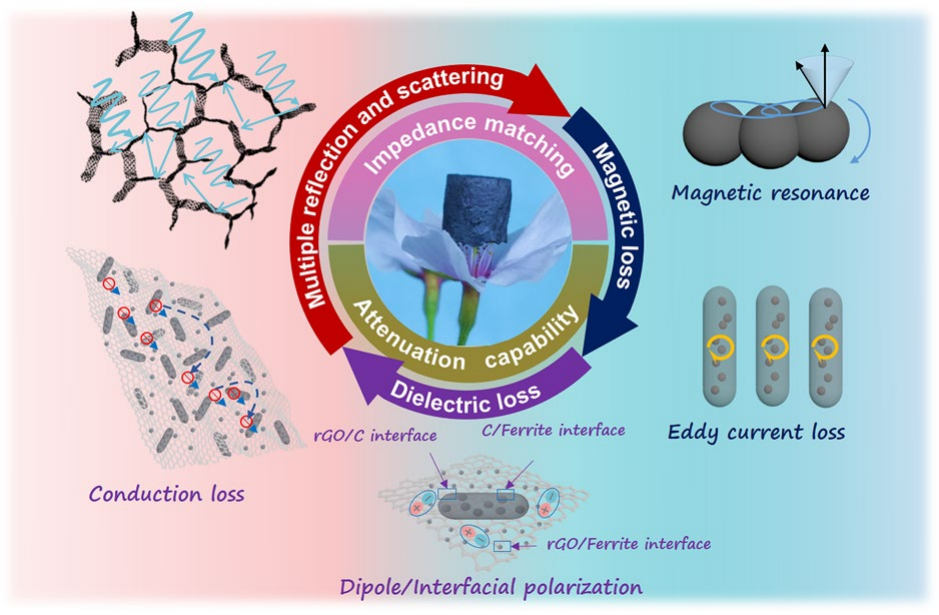

**Supplementary Information:**

The online version contains supplementary material available at 10.1007/s40820-022-00851-3.

## Introduction

High-performance microwave absorption (MA) materials exhibiting broadband microwave attenuation capability, a low density, and a low thickness, which can eliminate the adverse effects of electromagnetism on human health, electronic equipment, and military security, are in high demand [1–10]. In MA materials, the interest in nanostructures derived from metal–organic frameworks (MOFs) such as metals/metal compounds, carbon, and their composites, which are prepared via the high-temperature pyrolysis of MOF precursors is increasing because they show excellent electrical conductivity, good magnetism, and sufficient defects and interfaces, providing obvious merits in terms of both impedance matching and microwave loss [11–14]. Derivatives of MOFs are typically used as fillers for matrices to fabricate MA materials. Owing to their inherent properties such as well-dispersed nanoparticles and pristine microstructures at the nanoscale, MOF derivatives achieve good MA performance [15–19]. However, the practical application of MOF derivatives is hindered by challenges such as the high density, large loading content, and uncontrolled distribution in matrices. The direct fabrication of MOF derivatives with stable three-dimensional (3D) lightweight architectures and controllable length scales to achieve microwave absorbers is desirable yet a major challenge.

Graphene oxide (GO) has been considered an ideal precursor for assembling materials with extended architectures such as films, aerogels, or foams with MOF nanocrystals owing to its functional surface and large surface area [20–25]. The beneficial properties of individual compounds are retained in MOF/reduced GO (rGO) hybrid composites. The presence of graphene also enhances the electrical conductivity and mechanical properties of the composites. Moreover, 3D architectures can provide a hierarchical porous structure comprising nanoporous MOF derivatives and macroporous rGO aerogels, further extending the functional applications of MA materials [26]. Therefore, the integration of 3D graphene-based aerogels and MOF derivatives is a promising strategy for fabricating hierarchically porous high-performance MA materials with magnetic and dielectric synergy.

Generally, the fabrication of 3D MOF/graphene-based aerogels is divided into two categories: the in situ growth of MOFs on a preformed 3D graphene-based framework [23] and interfacial coordination between GO sheets and MOF precursors [24]. The former approach is an attractive strategy for preparing MOF/graphene-based aerogels with controlled 3D porous structures and uniformly distributed MOF particles on the graphene surface; however, the resultant interfacial binding is weak and this approach involves complicated synthetic steps. The interfacial coordination and assembly route for synthesizing 3D aerogels involve the direct synthesis of MOF particles in the presence of GO. The most straightforward technique for preparing MOF/graphene-based aerogel is the mixing of MOF precursors and GO at ambient temperature before heating the mixture under solvothermal or reflux conditions, which are necessary for MOF synthesis [25]. However, in this technique, excess MOF precursors must be introduced directly, possibly inducing the aggregation of GO nanosheets and the uncontrolled distribution of each component in the obtained MOF/rGO composite. Therefore, developing a convenient synthetic methodology for MOF/rGO hybrid aerogel materials is essential.

Here, we report a facile route for synthesizing MOF/rGO hybrid aerogels based on the direct gelation of GO initiated using MOF crystals in an aqueous solution. In a typical procedure, MIL-88A nanorods are introduced in a GO aqueous dispersion to eliminate the electrostatic repulsive forces between GO nanosheets, enabling the linking of GO nanosheets to form a stable 3D hydrogel under moderate heating conditions. The use of metal ions exposed on the surface of one-dimensional (1D) MIL-88A nanorods and functioning as joining sites enhances the linking of GO nanosheets to form a 3D network because of metal–oxygen covalent and electrostatic interactions. The gelation process is easy and does not involve any complicated synthetic step and the use of additional chemical reagents. Subsequently, we reveal the impressive MA performance of the prepared MOF/rGO-derived magnetic and dielectric aerogels Fe_3_O_4_@C/rGO and Ni-doped Fe_3_O_4_@C/rGO aerogels.

## Experimental Section

### Synthesis of MIL-88A Nanorods

In a typical synthesis, 2.1638 g FeCl_3_·6H_2_O (8 mmol) was dispersed into 20 mL deionized water by magnetic stirring for 15 min. Then 0.9288 g (8 mmol) fumaric acid was dispersed into 50 mL deionized water under stirring until the solution became clear. These solutions were then mixed in a 100 mL Teflon-lined autoclave. After stirring for 15 min, the autoclave was transferred to normal oven and heated at 100 °C for 4 h to obtain MIL-88A. At last, the products were filtered and washed with ethanol and water for three times and finally dried at 60 °C overnight.

### Synthesis of Ni-doped MIL-88A Nanorods

For the preparation of Ni-doped MIL-88A nanorods, only the species and content of metal ions were changed. Specifically, 1.0812 g FeCl_3_·6H_2_O (4 mmol) and 1.1631 g Ni(NO_3_)_2_·6H_2_O (4 mmol) were employed for Ni-doped MIL-88A.

### Preparation of MOF/rGO Aerogels

The MOF/rGO aerogels were synthesized as follows. Typically, 1 mL of 10 mg mL^−1^ MIL-88A aqueous solution was dispersed into 2 mL dispersion of GO (5 mg mL^−1^) by shaking for 1 min, standing for 1 min and repeating 3 times, respectively. The solutions were then heated at 95 °C for 5 h in an oven to form hydrogels. At last, the hydrogels were frozen for 24 h and freeze-dried for 24 h to obtain MIL-88A/graphene aerogels. Other Ni-doped MIL-88A/graphene aerogels were synthesized by the same way.

### Preparation of Pea-like Fe_3_O_4_@C/rGO Aerogels

For the preparation of pea-like Fe_3_O_4_@C/rGO aerogels, the MIL-88A/rGO aerogels were put into a glass tube and heated to 800 °C for 1 h in Ar with a heating rate of 3 °C min^−1^.

### Preparation of Cocoon-like Ni-doped Fe_3_O_4_@C/rGO Aerogels

For the preparation of cocoon-like Ni-doped Fe_3_O_4_@C/rGO, the Ni-doped MIL-88A/rGO aerogels were put into a glass tube and heated to 800 °C for 1 h in Ar with a heating rate of 3 °C min^−1^.

### Characterization

The microscopic and structure characterizations of aerogel samples were carried out through scanning electron microscopy (SEM, Zeiss Ultra-55), transmission electron microscope (TEM, FEI Tecnai G220 TWIN), X-ray photoelectron spectroscopy (XPS, Thermo Scientific K-Alpha), and X-ray powder diffraction (XRD, Rigaku Co., Cu Kα 1.5406 Å, 40 kV, 40 mA, D/teX Ultra 250 detector).

### MA Measurements

To measure the MA performance, MOF/rGO-derived magnetic-dielectric aerogels were impregnated with paraffin. Then the obtained composites were cut into concentric rings with an inner diameter of 3.04 mm and outer diameter of 7.00 mm. Electromagnetic parameters were measured using a coaxial method on a vector network analyzer (Agilent 5324A) in the frequency range of 2 − 18 GHz. The filling loading content of as-prepared aerogels in paraffin wax matrix can be calculated as follows:

1 $$w = \rho_{{{\text{aerogel}}}} \times {{v_{{{\text{ring}}}} } \mathord{\left/ {\vphantom {{v_{{{\text{ring}}}} } {m_{{{\text{ring}}}} }}} \right. \kern-\nulldelimiterspace} {m_{{{\text{ring}}}} }}$$

where *ρ*_aerogel_ is the density of aerogel, *v*_ring_ and *m*_ring_ is the volume and the weight of the ring, respectively.

### RCS Simulation

COMSOL Multiphysics 5.6 was used for simulating the radar cross-sectional of the MOF/rGO-derived magnetic-dielectric aerogels. According to metal back model, the simulation model of the specimens was established as a square (10×10 cm^2^) with dual layers, which consisted of an aerogel absorption layer and a back plate of the perfect electric conductor (PEC). The thickness of the bottom PEC layer was 1.0 mm, and the absorber layer thickness values were set as 2.5 and 3.5 mm at the frequency of 15 and 10 GHz, respectively. The aerogel-PEC model plate is placed on the X-O-Y plane, and linear polarized plane electromagnetic waves incident from the positive direction of the *Z* axis to the negative direction of the *Z*-axis. Meanwhile, the direction of electric polarization propagation is along the *X*-axis. Open boundary conditions are setting in all directions with field monitor frequency of 10 and 15 GHz. The RCS values can be described as follows:

2 $$\sigma \left( {dB\;{\text{sm}}} \right) = 10\log \left( {\left( {{{4\pi S} \mathord{\left/ {\vphantom {{4\pi S} {\lambda^{2} }}} \right. \kern-\nulldelimiterspace} {\lambda^{2} }}} \right)\left| {{{E_{s} } \mathord{\left/ {\vphantom {{E_{s} } {E_{i} }}} \right. \kern-\nulldelimiterspace} {E_{i} }}} \right|} \right)^{2}$$

Here, *S* is the area of the target object simulation model, *λ* is the wavelength of electromagnetic wave, *E*_*s*_ and *E*_*i*_ represent the electric field intensity of scattered wave and the incident wave, respectively.

## Results and Discussion

### Synthesis of MOF/rGO-derived Aerogels and Their Gelation Mechanism

Fe_3_O_4_@C/rGO and Ni-doped Fe_3_O_4_@C/rGO magnetic and dielectric aerogels were prepared using a three-step procedure involving the synthesis of MOFs, MOF/rGO aerogels, and MOF/rGO-derived magnetic and dielectric aerogels. To fabricate MIL-88A nanorods, FeCl_3_·6H_2_O was first mixed with fumaric acid (organic ligand) in an aqueous solution. The resulting mixture was then subjected to hydrothermal treatment at 100 °C. By changing the composition of the metal ions (e.g., Ni: Fe ratio of 1:1 in the reactants), Ni-doped MIL-88A nanorods were obtained. The morphology of the as-prepared MIL-88A and Ni-doped MIL-88A nanorods was characterized using SEM. The SEM images of both the MIL-88A and Ni-doped MIL-88A nanorods reveal highly uniform hexagonal rod-like nanostructures (Figs. S1a, b and S2a, b). Based on the elemental mapping images of MIL-88A, Fe, C, and O are homogeneously distributed in the hexagonal nanorods (Fig. S1c–e). An additional elemental Ni is also detected in the Ni-doped MIL-88A sample (Fig. S2c–f). After introducing Ni, the average length and width of the hexagonal nanorods decrease from 3.3 to 1.9 μm and 650 to 550 nm, respectively (Fig. S3), indicating a change in the growth rate and crystal size. Furthermore, the doping process did not considerably modify the crystal structure and composition of MIL-88A [27], which was confirmed from its XRD patterns (Fig. S4).

**Fig. 1:**
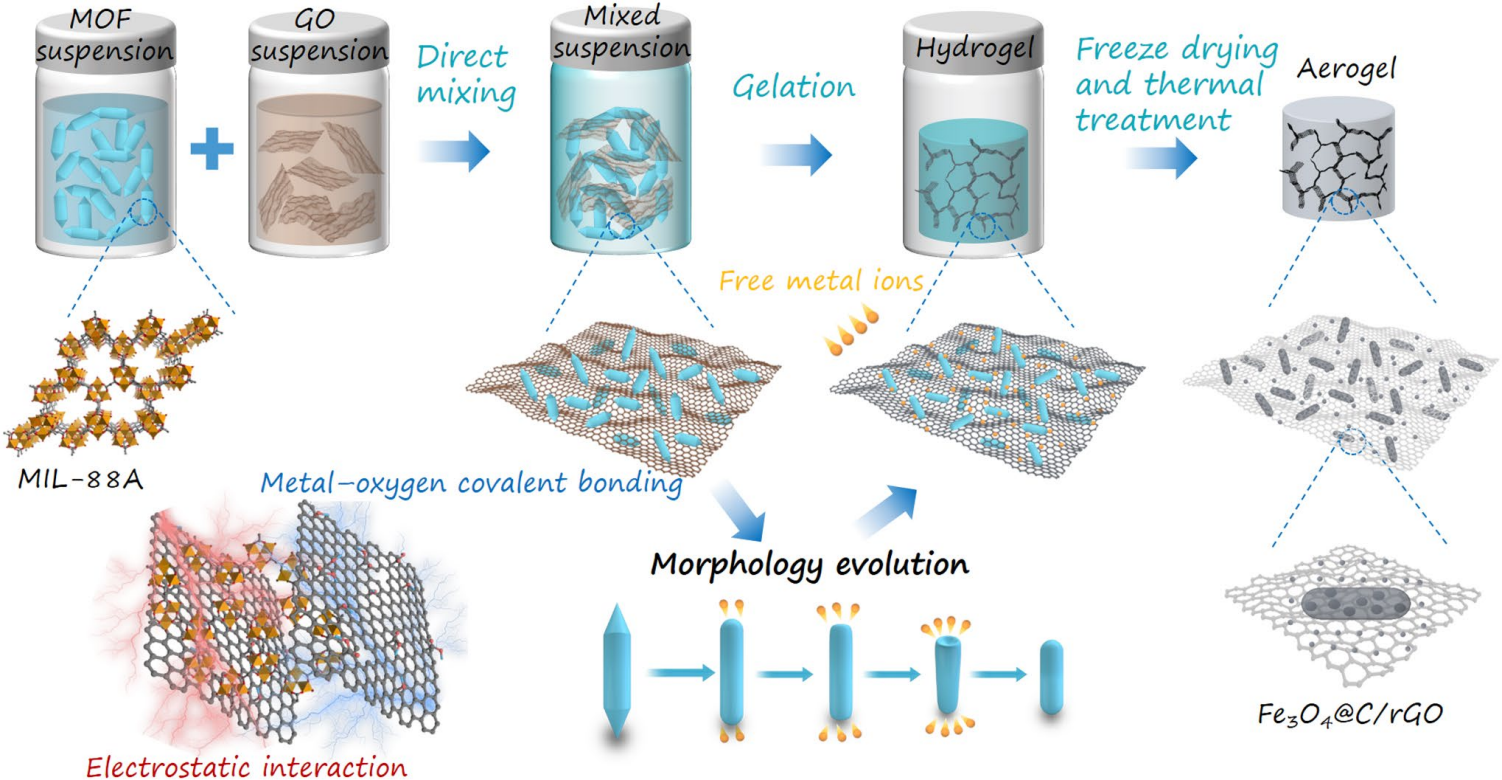
Schematic of the fabrication process of MOF/rGO hybrid aerogels by the use of MOFs to directly initiate the gelation of GO strategy

**Fig. 2:**
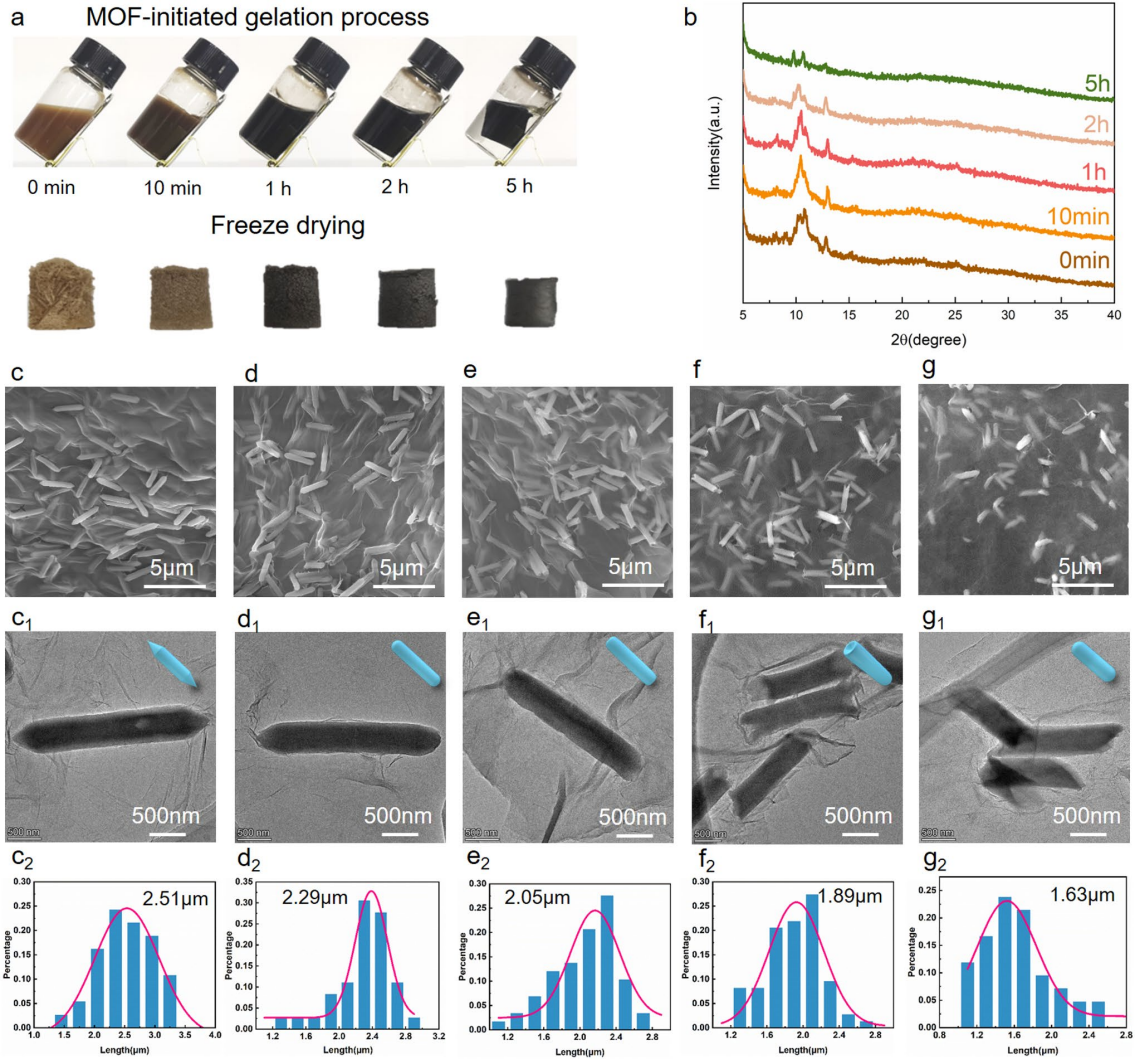
Representative **a** images during gelation and freeze drying, **b** XRD patterns, and **c–g** rod length distributions based on the SEM and TEM images of MIL-88A/rGO aerogels prepared at different gelation times

Figure 1 schematically presents the preparation processes of 3D MOFs/rGO aerogels and their derived aerogels. In brief, a GO aqueous suspension and a presynthesized MOF crystal suspension (e.g., containing MIL-88A) were mixed under vigorous shaking conditions. Owing to the metal–oxygen covalent or electrostatic interactions between the free Fe^3+^ on the MOF crystal surfaces and the oxygenated functional groups (i.e., –OH and –COOH) of GO, the precipitation of insoluble MIL-88A nanorods could be prevented in the GO nanosheets, thus affording stable suspensions. The mixed suspensions were rapidly transformed into wet gels in 10 min under moderate heating conditions. Under prolonged gelation time, the color of the gels gradually changed from brown to black, accompanied by volume shrinkage (Fig. 2a). This suggests that GO can be sufficiently reduced using MIL-88A without the use of additional chemicals or reagents.

To further explore the MOF-mediated gelation process, the evolution of the crystal structure and morphology of MIL-88A/rGO aerogels prepared at different gelation times was investigated (Fig. 2a). In the XRD patterns (Fig. 2b), the peak intensity gradually decreases and only two subpeaks of the (101) and weak (200) crystallographic facets are retained. The MIL-88A nanorods were randomly coupled with rGO nanosheets (Fig. 2c–g). The average length of the nanorods decreased from 2.5 to 1.6 μm (Fig. 2c2–g2). Moreover, the shape of the nanorod ends transformed from a hexagonal cone to a dome. Subsequently, nanorods with shortened ends were formed, finally producing small nanorods with dome-like shapes (Fig. 2c1–g1). During the gelation process, the MIL-88A nanorods were though to decompose and coordinate with the oxygenated groups of GO at both ends of the hexagonal structures, producing small nanorods anchored on the GO sheets and subsequently affording stable gels.

Therefore, the MIL-88A-mediated gelation of GO is divided into two steps. In the first step, the electrostatic interaction between free Fe^3+^ and the functional groups on a GO surface destroy the electrostatic repulsive forces between the GO nanosheets. In the second step, the crosslinking of GO nanosheets is assisted by Fe^3+^ functioning as linkers, promoting the stacking of GO nanosheets and initiating the assembly of the sheets into a 3D network. Such a gelation process is easy to achieve and does not involve any complicated synthetic step and the use of additional reagents. Furthermore, aerogels with different weight ratios of MOFs and GO were prepared by adjusting the initial concentrations of MOFs in mixed suspensions. MIL-88A/rGO and Ni-doped MIL-88A/rGO aerogels with different MOFs/GO weight ratios were obtained (Figs. S5, S6), which show uniform cylindrical structures from top to bottom, indicating the homogeneous distribution of MOFs in the aerogels without precipitate formation. At a low weight ratio of MOFs and GO of 1:10, a low-shrinkage aerogel was achieved owing to insufficient crosslinkers (i.e., Fe^3+^) in the reaction mixture.

**Fig. 3:**
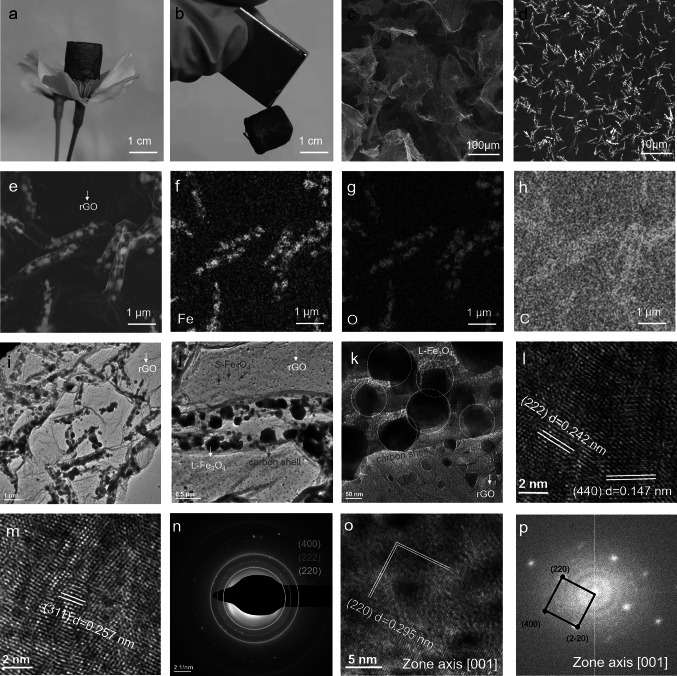
**a**, **b** Optical, **c**–**e** SEM, **f**–**h** elemental mapping (Fe, O, and C), **i**–**k** TEM, **l**, **m**, **o** HRTEM, and **n**, **p** SEAD and fast Fourier transform images of **m** and **o**, respectively, of the Fe_3_O_4_@C/rGO aerogel

Under an additional thermal treatment, the MIL-88A/rGO and Ni-doped MIL-88A/rGO aerogels were converted to Fe_3_O_4_@C/rGO and Ni-doped Fe_3_O_4_@C/rGO magnetic and dielectric aerogels, respectively, showing density values of 6.2 and 5.3 mg cm^−3^, respectively. The Fe_3_O_4_@C/rGO aerogel is ultralight and exhibits magnetic properties (Fig. 3a, b). Furthermore, it presents highly porous 3D structures with microscale pores, which are obtained via the interlinking of the rGO sheets (Fig. 3c). Pea-like core–shell nanocapsules, which were derived from the MIL-88A nanorods, are uniformly coated on the surface of the rGO sheets (Fig. 3d). These nanocapsules comprise a carbon shell and large Fe_3_O_4_ (L-Fe_3_O_4_) cores (Fig. 3e–h). Similar pea-like core–shell nanostructures were observed in the TEM images of samples containing rGO nanosheets (Fig. 3i–k). The high-resolution TEM (HRTEM) of the L-Fe_3_O_4_ nanoparticles reveals lattice distances of 0.147, 0.242, and 0.257 nm (Fig. 3l–m), corresponding to the (440), (222), and (311) planes of Fe_3_O_4_. The selected-area electron diffraction (SAED) pattern (Fig. 3n) also confirms the transformation of MIL-88A to Fe_3_O_4_. Furthermore, the HRTEM (Fig. 3o) and corresponding FFT (Fig. 3p) patterns show Fe_3_O_4_ nanoparticles with sizes of < 50 nm anchored on the rGO surface (Fig. 3j–k), suggesting that the Fe^3+^ coordinated with the oxygenated groups of rGO was converted to Fe_3_O_4_ during high-temperature annealing.

**Fig. 4:**
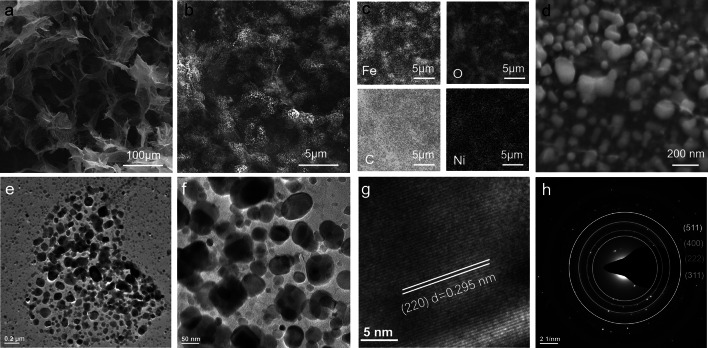
**a, b, d** SEM, **c** elemental mapping (Fe, O, C, and Ni), **e, f** TEM, **g** HRTEM, and **h** SEAD images for **f** of the Ni-doped Fe_3_O_4_@C/rGO aerogel

**Fig. 5:**
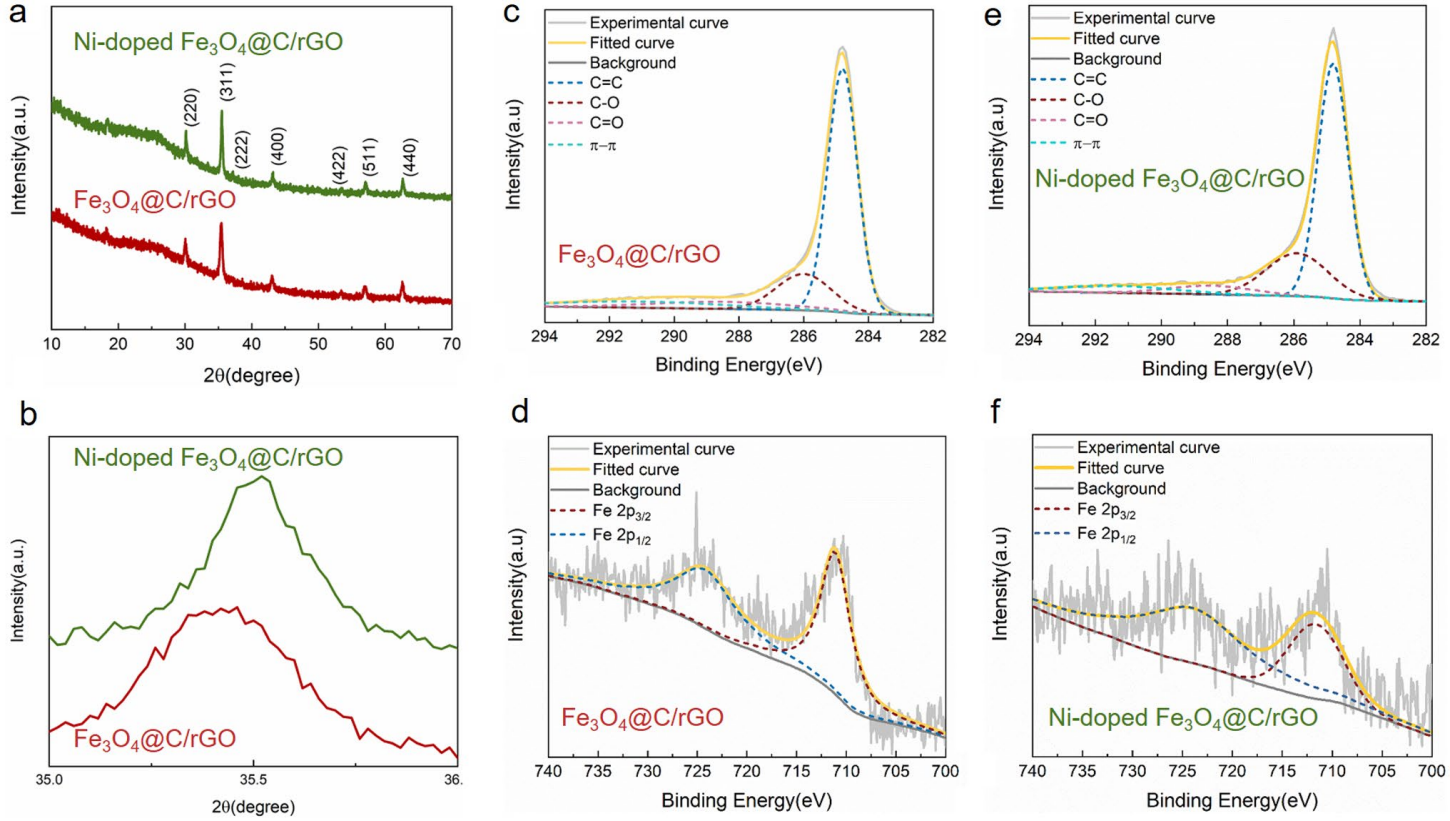
**a-b** XRD patterns and **c**–**f** high-resolution XPS spectra of **c, e** C 1* s* and **d, f** Fe 2*p* of Fe_3_O_4_@C/rGO and Ni-doped Fe_3_O_4_@C/rGO aerogels

In the case of the Ni-doped Fe_3_O_4_@C/rGO aerogel, the inner morphology also shows a 3D interconnected porous microstructure (Fig. 4a) with numerous cocoon-like core–shell nanocapsules on rGO walls (Fig. 4b). The elemental mapping images of the Ni-doped Fe_3_O_4_@C/rGO aerogel (Fig. 4c) show the distribution of Fe, O, C, and Ni in the rGO sheets. The enlarged SEM and TEM images further confirm the presence of cocoon-like nanocapsules, which consist of a thin shell and nanoparticle cores (Fig. 4d–f). The magnified HRTEM image (Fig. 4g) reveals lattice fringes with a spacing distance of 0.295 nm, corresponding to the (220) plane of Fe_3_O_4_. Clear diffraction rings are observed in the corresponding SAED pattern (Fig. 4h), where the components are consistent with those observed in the XRD results (Fig. 5a).

The structures and composition of the Fe_3_O_4_@C/rGO and Ni-doped Fe_3_O_4_@C/rGO aerogels were further examined using XRD and XPS. The XRD patterns of the aerogels exhibit the characteristic peaks of Fe_3_O_4_ and a broad peak at 2*θ* = 26°, corresponding to the (002) reflection of rGO sheets (Fig. 5a). However, a tiny shift in the diffraction peaks of the Ni-doped Fe_3_O_4_/rGO aerogel is observed (Fig. 5b). Based on the Bragg laws, an increase in 2*θ* corresponded to a decrease in the d-spacing, which indicates a reduction in the magnitude of the lattice parameters. During pyrolysis, Ni^2+^ would enter the Fe_3_O_4_ lattice and replace Fe^2+^, decreasing the lattice parameter because the ionic radius of Ni^2+^ (0.069 nm) is smaller than that of Fe^2+^ (0.074 nm) [28, 29]. This phenomenon is also observed in the case of pure Ni-doped MIL-88A nanorod–derived nanomaterials (Fig. S7).

The high-resolution C 1*s* spectra of the aerogels (Fig. 5c, e) reveal binding energies of 284.6, 285.8, 288.2, and 289.8 eV, representing the C–C, C–O, C = O, and *π*–*π* interactions, respectively, thus indicating a sufficient reduction of GO [30, 31]. The high-resolution Fe 2*p* spectra of the aerogels (Fig. 5d, f) show two obvious peaks at binding energies of 711.2 and 724.8 eV, representing Fe 2*p*_3/2_ and Fe 2*p*_1/2_, respectively, thereby indicating the presence of Fe_3_O_4_ [32, 33]. Ni is not detected in the XPS pattern of the Ni-doped Fe_3_O_4_@C/rGO aerogel, ascribed to its trace amount. The ICP measurements reveals Ni (trace amount: 0.02 wt%) in the pure Ni-doped MIL-88A–derived Fe_3_O_4_@C nanomaterial.

### MA Performance

**Fig. 6:**
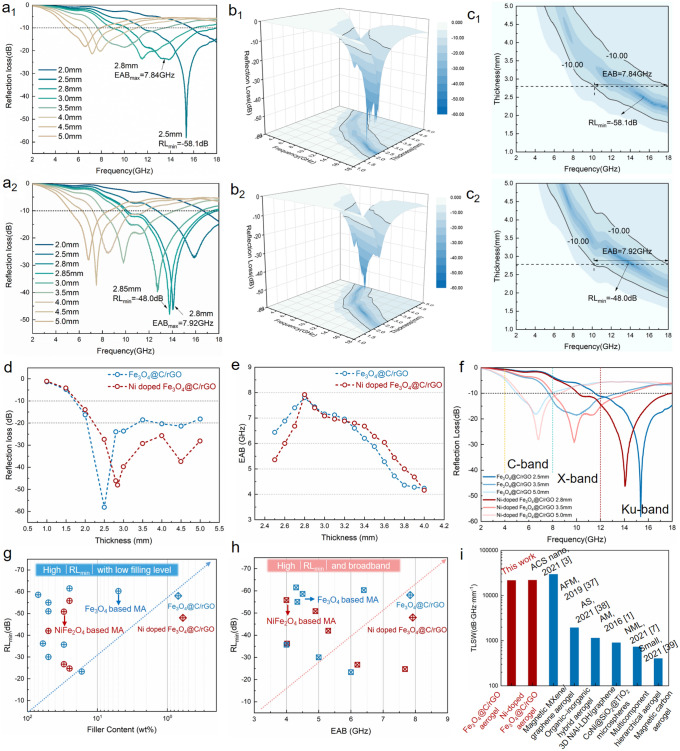
**a** RL − *f* curves, **b** 2D, and **c** 3D representations of the *RL* values. **d** *RL*_min_ and **e** EAB values at different thicknesses. **f** Selected *RL*–f curves at different wavebands. **g**, **h** Comparison of the EMA performance considering the *RL*_min_, filler content, and EAB with reported spinel structured MFe_2_O_4_ (M = Fe and Ni) composites. **i** TLSW values of the reported representative EMA materials

Aerogel/paraffin composites were fabricated by immersing the as-prepared aerogels in molten paraffin to retain the 3D porous structure (Fig. S8), instead of mixing rGO aerogel powders with paraffin to avoid the possible agglomeration and uncontrollable distribution of aerogels. The reflection loss (RL) curves and the two-dimensional (2D) and 3D representations of the RL for the Fe_3_O_4_@C/rGO and Ni-doped Fe_3_O_4_@C/rGO aerogels (Fig. 6a–c) are calculated based on the transmission line theory using Eqs. 3 and 4 [34]:

3 $${\text{RL}} = 20\lg \left| {\frac{{Z_{{{\text{in}}}} - Z_{0} }}{{Z_{{{\text{in}}}} - Z_{0} }}} \right|.$$

4 $$Z_{{{\text{in}}}} = Z_{0} \sqrt {\frac{{\mu_{r} }}{{\varepsilon_{r} }}} \tanh \left( {j\frac{{2\pi fd\sqrt {\mu_{r} \varepsilon_{r} } }}{c}} \right)$$

where Z_in_, Z_0_, c, and f denote the normalized input impedance of the absorber, impedance of air, velocity of light, and frequency of microwaves, respectively. Furthermore, ε_r_, µ_r_, and d represent the relative complex permittivity, relative complex permeability, and thickness of the absorber, respectively.

Based on Figs. 6a1, a2 and S9, the minimum *RL* value (RL_min_) of the optimal Fe_3_O_4_@C/rGO aerogel reaches − 58.1 dB at 15.4 GHz at a thickness of 2.5 mm and the effective absorption bandwidth (EAB) ranges from 10.16 to 18.0 GHz at a thickness of 2.8 mm, covering the entire Ku band (12–18 GHz). In the case of the Ni-doped Fe_3_O_4_@C/rGO aerogel, the strong absorption peak shifts to a low-frequency range with increasing matching thickness of the sample, subsequently reaching an RL_min_ of − 48.0 dB at 14 GHz at a thickness of 2.85 mm. Alternatively, the RL_min_ of the Ni-doped Fe_3_O_4_@C/rGO aerogel exceeds − 20 dB at thicknesses of 2.5–5 mm (Fig. 6f) and the EAB range from 10.08 to 18 GHz, with the maximum reaching 7.92 GHz, covering the entire Ku band and 48% of the *X* band (8–12 GHz) at a thickness of 2.8 mm. The Fe_3_O_4_@C/rGO and Ni-doped Fe_3_O_4_@C/rGO aerogels show efficient electromagnetic wave absorption in the entire *X* band and 75% of the C band at thicknesses of 3.5 and 5 mm, respectively (Fig. 6d). These results confirm the considerable effect of the size and composition of the original MIL-88A nanorods on the EM response capability, thus affecting the resulting MA performance.

Based on the aforementioned results, the Fe_3_O_4_@C/rGO and Ni-doped Fe_3_O_4_@C/rGO aerogels show remarkable MA performance based on their high RL_min_ and wide EAB values at low thicknesses (− 58.1 dB, 6.48 GHz, and 2.5 mm and − 46.2 dB, 7.92 GHz, and 2.8 mm, respectively) with ultralow filling contents (0.7 and 0.6 wt%). The filling content and EAB for the ultralight magnetic and dielectric aerogels are compared with corresponding values (Tables S1 and S2) reported in the recent literature on spinel structured MFe_2_O_4_ (M = Fe and Ni) composites [35] and rGO aerogel–based microwave absorbers [36]. As shown in Figs. 6g-h and S10, both the Fe_3_O_4_@C/rGO and Ni-doped Fe_3_O_4_@C/rGO aerogels present obvious advantages such as ultralow filling contents and broadband MA. Furthermore, to access a comprehensive MA performance with thick (*T*), light (*L*), strong (*S*), and wide (*W*) features, the TLSW value is obtained using the following equation of a material: |RL| (dB) × bandwidth (GHz)/thickness (mm)/filling ratio (wt%). Because the Fe_3_O_4_@C/rGO and Ni-doped Fe_3_O_4_@C/rGO aerogels achieve high TLSW values (Table S3 and Fig. 6i), these magnetic and dielectric aerogels can be categorized in the group of state-of-the-art MA materials such as magnetic MXene/graphene aerogel [3], organic–inorganic hybrid aerogel [37], 3D NiAl-LDH/graphene [38], CoNi@SiO_2_@TiO_2_ microspheres [1], multicomponent hierarchical aerogels [7], and magnetic carbon aerogel [39].

**Fig. 7:**
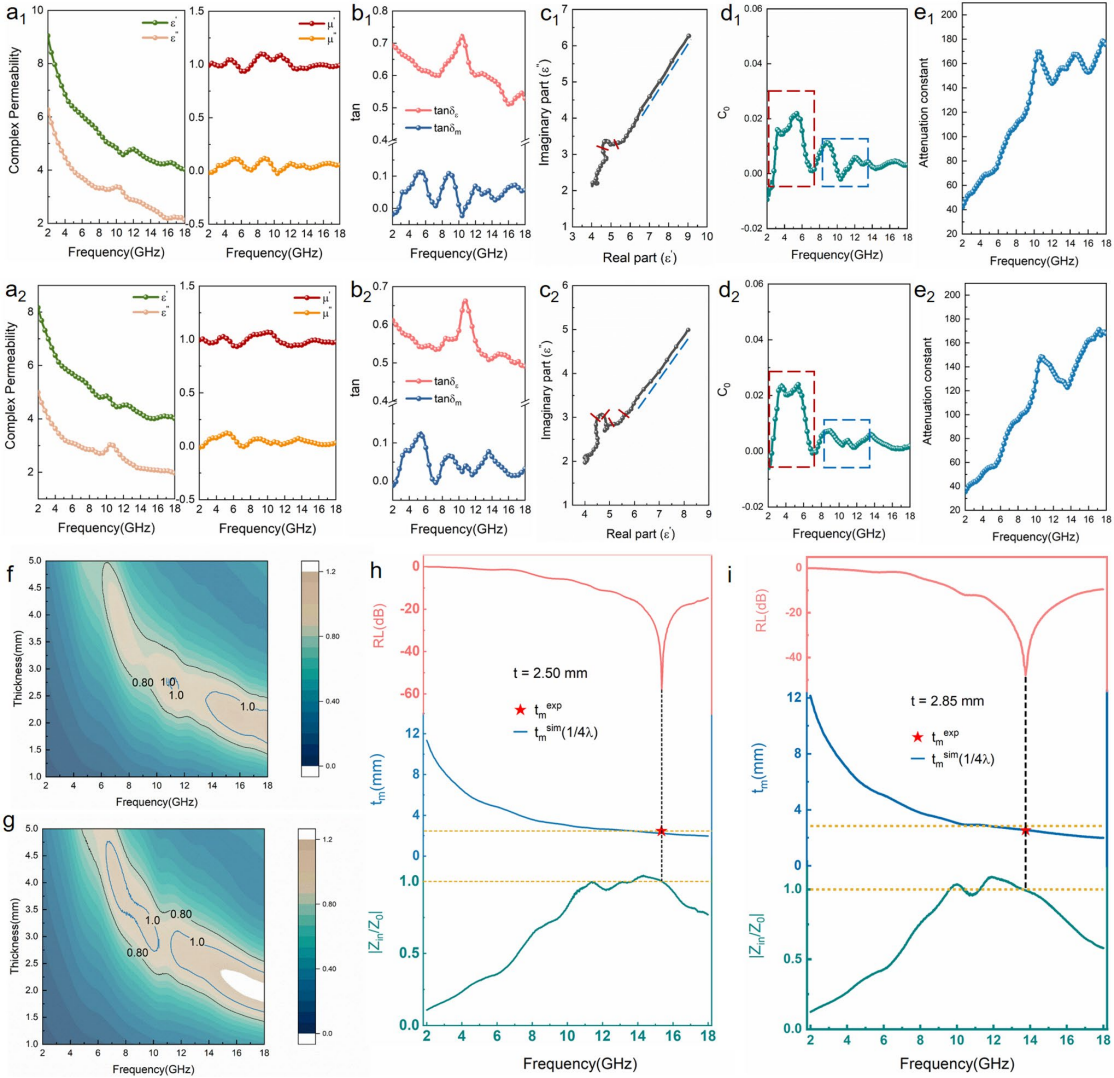
**a** Complex permittivity and permeability, **b** dielectric and magnetic loss tangents (tan*δ*_*ε*_ and tan*δ*_*μ*_, respectively), **c** Cole–Cole curves (*ε*′–*ε*″ plots), **d** *C*_0_–*f* curves, **e** attenuation constant (α), **f**, **g** impedance matching (*Z*_in_/*Z*_0_) and **h**, **i** RL/*t*_m_/*Z* − *f* curve for the prepared Fe_3_O_4_@C/rGO and Ni-doped Fe_3_O_4_@C/rGO aerogels

To clarify the MA mechanism, the electromagnetic parameters including the real (*ε*′ and *µ*′) and imaginary parts (*ε*″ and *µ*″) are shown in Figs. 7a and S11. *ε*′ and µ′ represent the storage capability of electric and magnetic energies, respectively, and *ε*″ and *µ*″ denote the dissipation capability of electric and magnetic energies, respectively. Generally, the dielectric loss of graphene-based aerogels is attributed to polarization relaxation and conduction losses (*ε*_*p*_″ and i″, respectively), which are further confirmed using the Cole–Cole curves of *ε*′–*ε*″ according to the Debye relaxation theory [40, 41]. The curves are divided into two parts: the part with several semicircles and a small *ε*′ value is related to the polarization loss and that with a straight line and a large *ε*′ value is related to the conduction loss (Fig. 7c). The conduction loss is ascribed to the migration of electrons in the 3D interconnected network structure [2, 40] and the carbon shells of the nanocapsules [34, 42, 43]. The multiple polarization relaxations in the Fe_3_O_4_@C/rGO and Ni-doped Fe_3_O_4_@C/rGO aerogels include the dipolar polarizations caused by the defects and functional groups on the rGO skeleton and multiple heterogeneous interfacial polarizations among Fe_3_O_4_@C or Ni-doped Fe_3_O_4_@C nanocapsules, small ferromagnetic nanoparticles, and rGO sheets [44, 45]. Moreover, based on doping engineering, the crystal structure of Ni-doped Fe_3_O_4_ can be modified and additional heterogeneous interfaces can be formed [46]. Compared with the pea-like Fe_3_O_4_@C nanocapsules, the cocoon-like Ni-doped Fe_3_O_4_@C nanocapsules shows highly maximized interfacial areas, reinforcing interfacial effects.

Because of the uniform incorporation of a ferromagnetic component from MIL-88A to the dielectric rGO aerogels, magnetic loss is also instrumental in the microwave attenuation capacity of both the aerogels. The *µ*′ and *µ*″ values of both the aerogels stable in the range of 0.91–1.12 and fluctuate in the range of − 0.05–0.15 (Fig. 7a). The negative *μ*″ value indicates that the magnetic energy from the induced magnetic field of the materials is transformed into the electric energy. Similar results have been observed in other magnetic carbon absorbers [47]. Generally, the magnetic loss is attributed to the eddy current and magnetic resonance loss (natural and exchange resonances) [3]. The eddy current loss is evaluated using Eqs. 5 and 6 [11].

5 $$\mu^{\prime \prime} \approx \frac{2}{3}\pi \mu_{0} \mu^{{\prime}{2}} \sigma d^{2} f$$

6 $$C_{0} = \mu^{\prime \prime } \mu^{\prime - 2} f^{ - 1} = \frac{2}{3}\pi \mu_{0} \sigma d^{2}$$

where μ_0_ denotes the vacuum permeability. *C*_0_ is positively correlated with *d*^2^ and *σ* (conductivity). The *C*_0_ value is almost stable in the range of 14–18 GHz in the *C*_0_ − *f* curves (Fig. 7d), indicating a magnetic loss from the eddy current loss. Alternatively, the large vibration area (blue dotted frame) of *C*_0_ at low frequencies of 2–7 GHz and low fluctuation area (red dotted frame) at high frequencies of 8–14 GHz are dominated by natural and exchange resonances, respectively [3]. Furthermore, the increased weight ratio of MOFs and rGO (3:2) induces the agglomeration of MOF derivatives (Fig. 12), affording reduced magnetic loss (Fig. S11c, d).

In addition to the intrinsic microwave attenuation capacity (Fig. 7e), impedance matching is another factor for evaluating the prepared high-efficiency absorbers [11, 12]. Impedance matching is used to characterize the degree of matching between the input impedance of the material and the impedance of a free space. If the impedance matching value (*Z* =|*Z*_in_/*Z*_0_|) is ~ 1, additional electromagnetic waves enter the absorbers; otherwise, electromagnetic waves are reflected from the material surface. The impedance matching values in a moderate region of 0.8–1.2 for both aerogels increased (Fig. 7f, g), affording improved electromagnetic wave loss performance.

The relation between the *RL*, thickness, and frequency can be obtained using the one-quarter wavelength model. The simulated thickness (*t*_*m*_) of an absorber at 2–18 GHz is estimated using Eq. 7 [15]:

7 $$t_{m} = \frac{{{\text{nc}}}}{{4f_{m} \sqrt {\left| {\mu_{r} } \right|\left| {\varepsilon_{r} } \right|} }}$$

where *n* and *f*_m_ denote the refractive index of the material and the matched frequency, respectively. When the *t*_m_ value is approximately consistent with the experimental thickness of a material, the resulting MA shows practical applications. Figure 7h, i show the typical RL–*f*, *t*_m_–*f*, and |*Z*_in_/*Z*_0_|–*f* curves of the Fe_3_O_4_@C/rGO and Ni-doped Fe_3_O_4_@C/rGO aerogels at thicknesses of 2.50 and 2.85 mm, respectively. The RL_min_ values are obtained at a frequency where |*Z*_in_/*Z*_0_|= 1 and the *t*_m_^exp^ values exactly falls in the *λ*/4 curves.

**Fig. 8:**
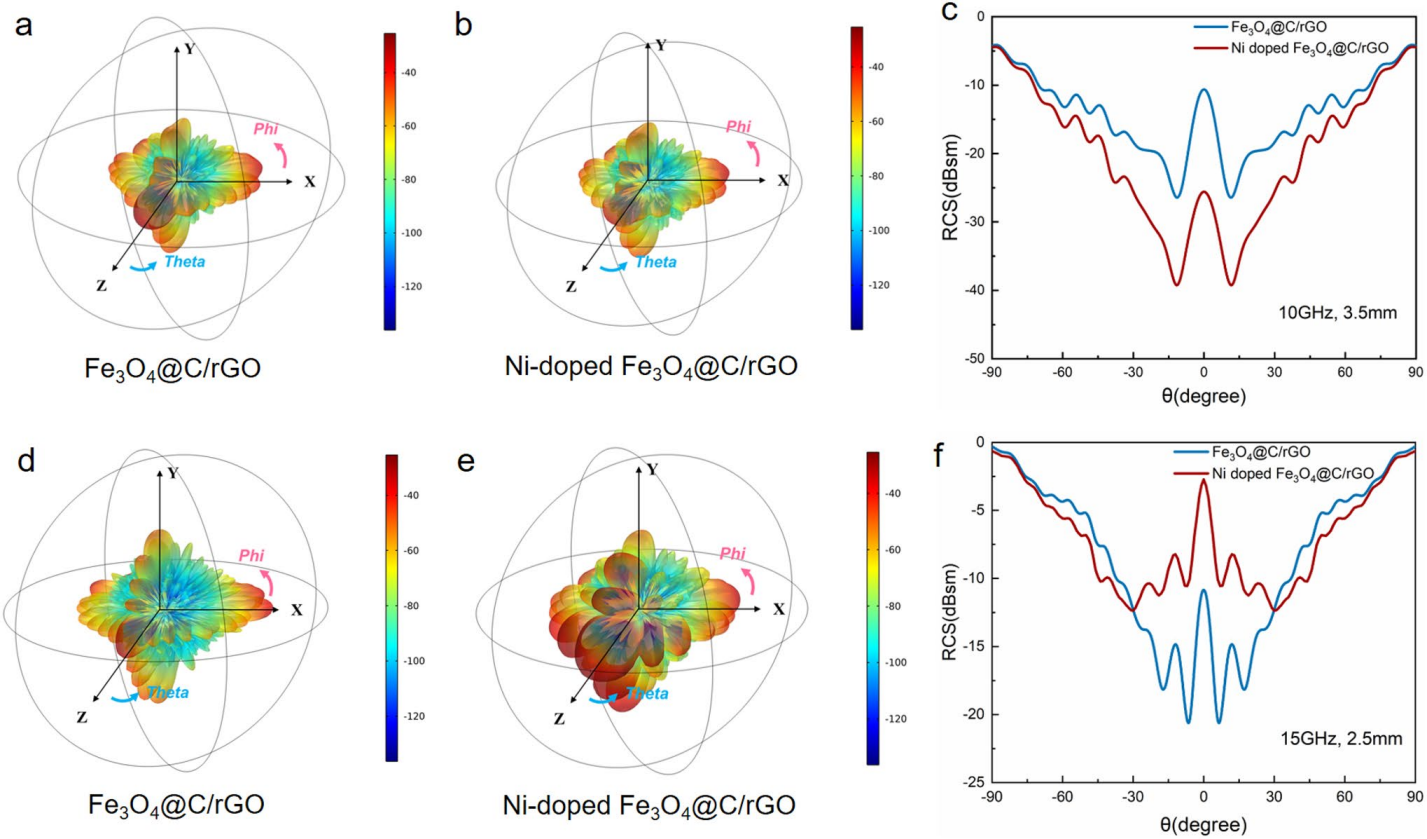
**a**, **b**, **d**, **e** 3D radar wave scattering signals, **c, f** RCS simulated curves of the prepared Fe_3_O_4_@C/rGO and Ni-doped Fe_3_O_4_@C/rGO aerogels

Figure 8 presents the CST simulation results for the Fe_3_O_4_@C/rGO and Ni-doped Fe_3_O_4_@C/rGO aerogels at 10 and 15 GHz, which reflect the real far-field conditions of MA performance [48]. At 10 GHz, both the aerogels exhibit weak scattering signals (Fig. 8a, b). The RCS value of the Ni-doped Fe_3_O_4_@C/rGO aerogel with a coating thickness of 3.5 mm is less than − 10 dB sm in the range of − 72° < *θ* < 72°, which is lower than that of the Fe_3_O_4_@C/rGO aerogel (Fig. 8c). The difference in the RCS values of the two aerogels is 15.1 dB sm at *θ* = 0°. At 15 GHz, the Ni-doped Fe_3_O_4_@C/rGO aerogel shows a stronger scattering signal than the Fe_3_O_4_@C/rGO aerogel (Fig. 8d, e). The RCS value of the Fe_3_O_4_@C/rGO aerogel is less than − 10 dB sm over the range of − 37° < theta < 37° at a coating thickness of 2.5 mm. These simulated results further confirm the enhanced MA performance of the Ni-doped Fe_3_O_4_@C/rGO aerogel in the *X* band. Furthermore, the Fe_3_O_4_@C/rGO aerogel is superior to the Ni-doped Fe_3_O_4_@C/rGO aerogel in terms of scattering signal at high frequencies, corresponding well with the MA properties of the former summarized in Fig. 6f.

### MA Mechanism

**Fig. 9:**
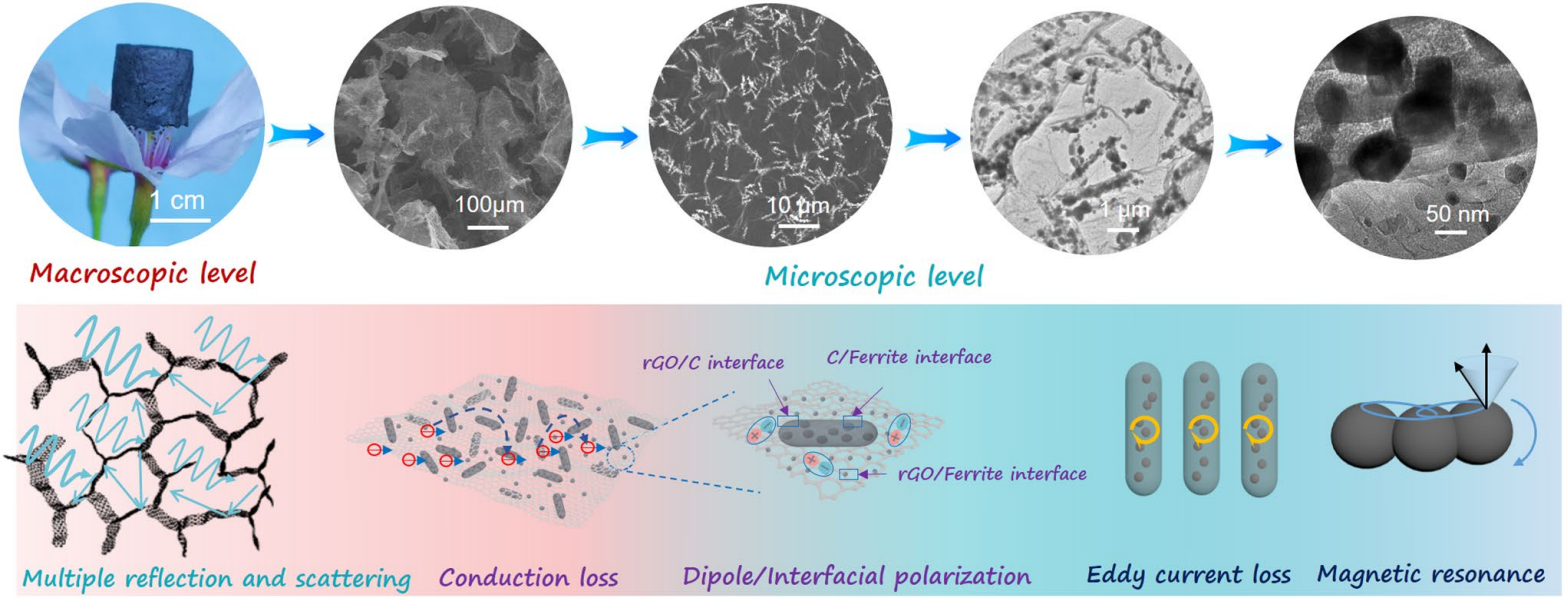
Schematic of the associated microwave absorption mechanism of the proposed MOF/rGO-derived magnetic and dielectric aerogels

Based on the aforementioned experiments and simulation analysis, the MA mechanism of the MOF/rGO-derived magnetic and dielectric aerogels is attributed to the synergistic effects of impedance matching and microwave attenuation capability (Fig. 9). At the macroscopic level, a 3D porous structure promotes the entry of electromagnetic microwaves into the aerogels instead of being reflected from the surface. Multiple random reflections and scatterings of electromagnetic waves occur repeatedly in microcellular free spaces, affording excellent impedance matching [40]. At the microscopic level, the synergistic dielectric and magnetic losses are crucial in the absorption attenuation mechanism of the MOF/rGO-derived magnetic and dielectric aerogels. The incident electromagnetic microwaves are captured and attenuated by the 3D multicomponent walls composed of the rGO nanosheets, Fe_3_O_4_@C or Ni-doped Fe_3_O_4_@C nanocapsules, and small ferromagnetic nanoparticles [18]. The multiple polarization relaxations in the aerogels include the dipolar polarizations caused by the defects and functional groups on the rGO skeleton and multiple heterogeneous interfacial polarizations of Fe_3_O_4_@C or Ni-doped Fe_3_O_4_@C nanocapsules, small ferromagnetic nanoparticles, and graphene sheets [12]. Moreover, the interconnected and conductive structure of the aerogels effectively promote the conduction loss. Furthermore, spatially dispersed ferromagnetic nanoparticles suspended within highly porous 3D frameworks afforded a multiscale magnetic network and could considerably contribute to the enhanced magnetic responding capacity [19].

## Conclusions

We demonstrated a green and convenient route for synthesizing MOF/rGO hybrid aerogels based on the gelation of GO in an aqueous dispersion directly initiated using MOF crystals. The gelation mechanism involves the elimination of the electrostatic repulsive forces at the joining sites provided by the free metal ions exposed on the surface of the MIL-88A nanorods, initiating the assembly of the sheets into a 3D network under moderate heating conditions. Such a gelation process is easy to achieve without complicated synthetic steps and additional chemicals or reagents. Furthermore, the compositions of the as-prepared aerogels can be precisely controlled by adjusting the initial concentrations of the MOF suspensions. Because of the good impedance matching and microwave attenuation capability, the proposed MOF/rGO-derived magnetic and dielectric aerogels show impressive MA performance. The Fe_3_O_4_@C/rGO and Ni-doped Fe_3_O_4_@C/rGO aerogels achieve strong RL_min_ (− 58.1 and − 46.2 dB, respectively) and broad EAB_max_ (6.48 and 7.92 GHz, respectively) with thicknesses of 2.5 and 2.8 mm and ultralow filling contents of 0.7 and 0.6 wt%, respectively. The CST simulation results also demonstrate that the prepared Ni-doped Fe_3_O_4_@C/rGO aerogel effectively suppresses the reflection and scattering of electromagnetic waves in the *X* band. Further, the Fe_3_O_4_@C/rGO aerogel shows good microwave attenuation capacity at high frequencies, thus providing a theoretical basis for practical far-field applications of the synthesized absorbers. Our findings provide guidance and inspiration for the design and fabrication of hierarchically porous MOF/rGO hybrid aerogels as MA materials for use in various fields.

## Supplementary Information

Below is the link to the electronic supplementary material.

Supplementary file1 (PDF 1816 KB)
