# Supplementary material for: Ultralight Magnetic and Dielectric Aerogels Achieved by Metal–Organic Framework Initiated Gelation of Graphene Oxide for Enhanced Microwave Absorption
Source: Nanomicro Lett. 2022 Apr 19;14:107. doi: 10.1007/s40820-022-00851-3 (PMC9019009; doi:10.1007/s40820-022-00851-3)
Supplement: Supplementary file 1 — Supplementary file1 (PDF 1816 KB) [file 40820_2022_851_MOESM1_ESM.pdf]

Supporting Information for

Ultralight Magnetic and Dielectric Aerogels Achieved by Metal-Organic Framework Initiated Gelation of Graphene Oxide for Enhanced Microwave Absorption

Xiaogu Huang[1, *], Jiawen Wei[1], Yunke Zhang[1], BinBin Qian[2, 5], Qi Jia[3], Jun Liu[3], Xiaojia Zhao[4], Gaofeng Shao[1, *]

[1] Institute of Advanced Materials and Flexible Electronics (IAMFE), School of Chemistry and Materials Science, Nanjing University of Information Science & Technology, Nanjing 210044, P. R. China

[2] Department of Chemical and Biological Engineering, Monash University, Victoria 3800, Australia

[3] College of Field Engineering, Army engineering university of PLA, Nanjing 210007, P. R. China

[4] Hebei Key Laboratory of Inorganic Nano-materials, College of Chemistry and Materials Science, Hebei Normal University, Shijiazhuang, 050024, P. R. China

[5] School of Chemistry and Environmental Engineering, Yancheng Teachers University, Yancheng, 224002, P. R. China

*Corresponding authors. E-mail: gfshao@nusit.edu.cn (Gaofeng Shao), hxg@nuist.edu.cn (Xiaogu Huang)

Supplementary Figures

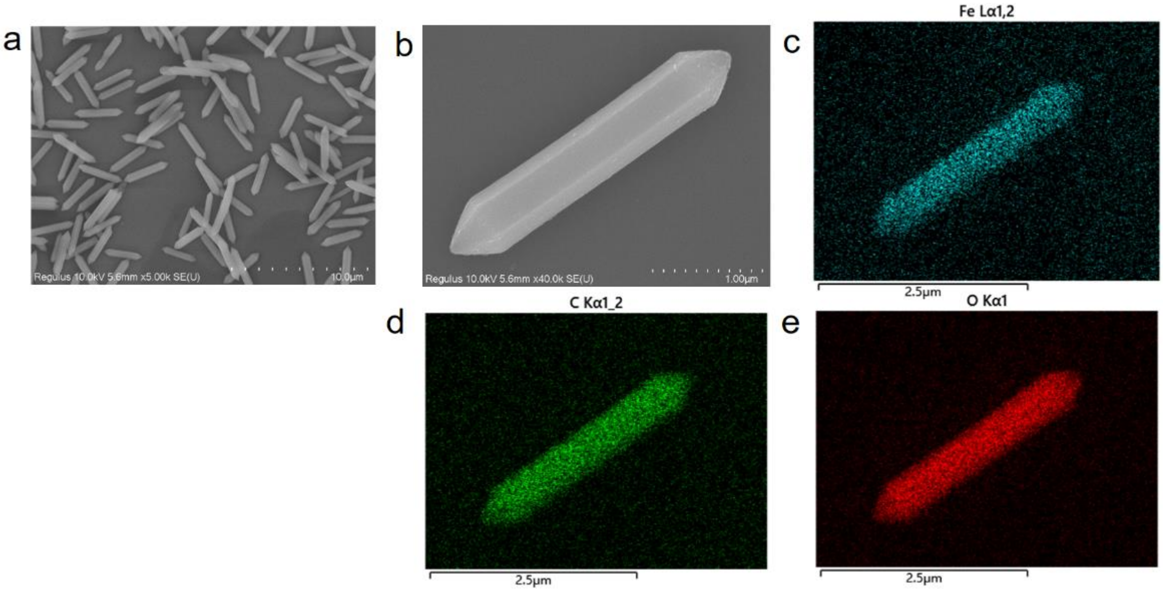

Fig. S1 Morphology and elemental mapping of MIL-88A

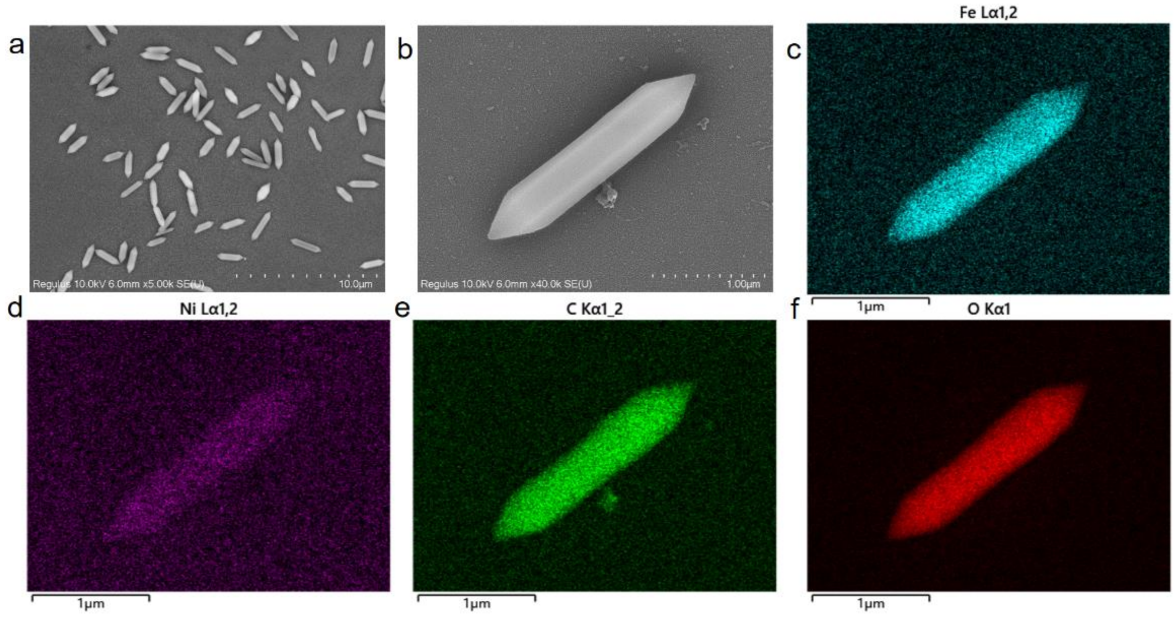

Fig. S2 Morphology and elemental mapping of Ni-doped MIL-88A

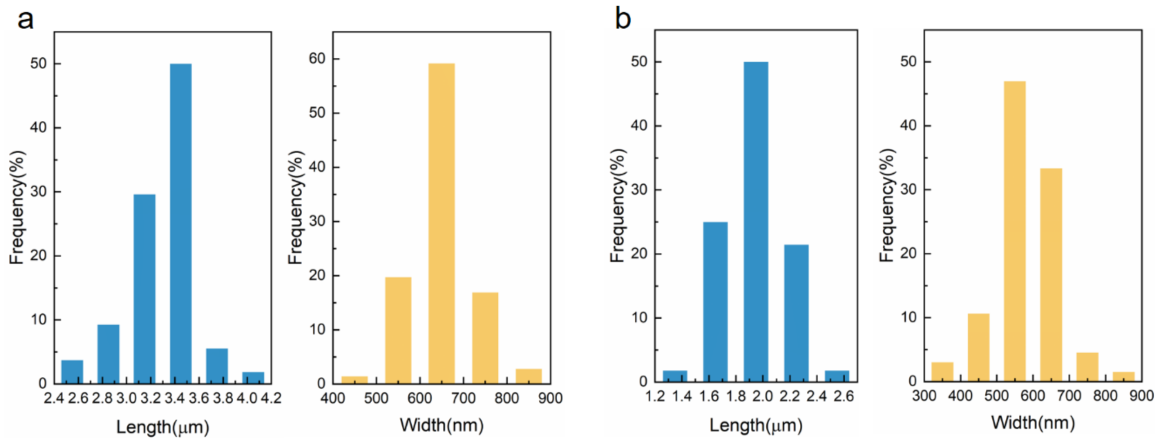

Fig. S3 Histograms for the rod length and width distribution of **a** MIL-88A and **b** Ni-doped MIL-88A

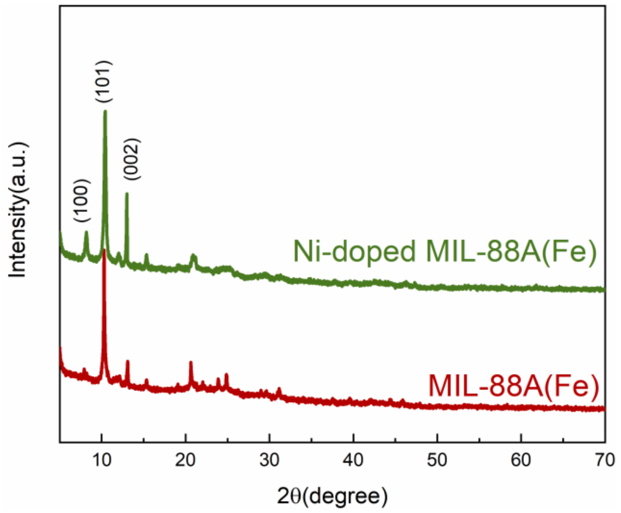

Fig. S4 XRD patterns of MIL-88A and Ni-doped MIL-88A

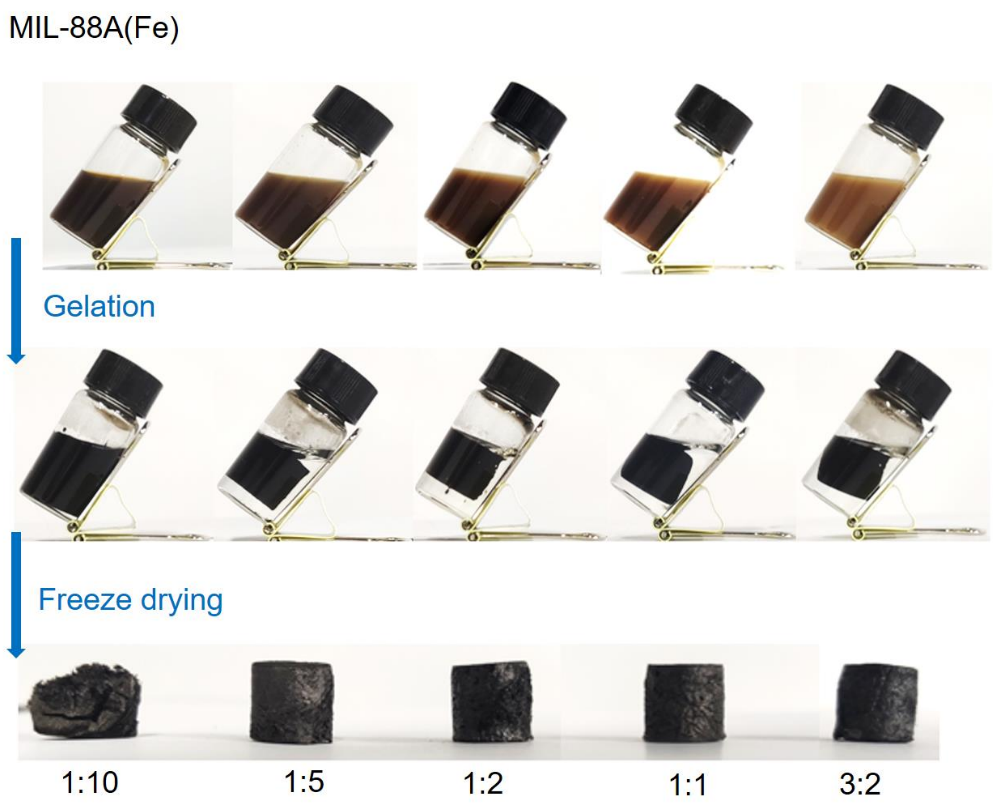

Fig. S5 Optical images of the fabrication process of MIL-88A/rGO aerogels with the different MIL-88A/GO weight ratios

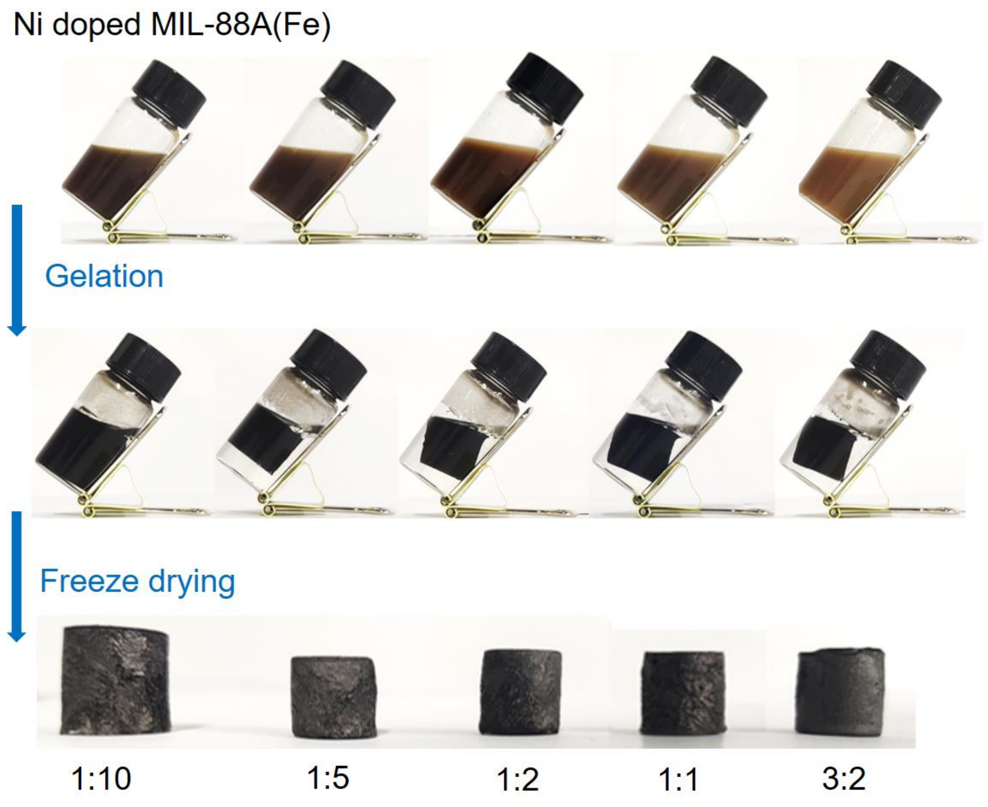

Fig. S6 Optical images of the fabrication process of Ni-doped MIL-88A/rGO aerogels with the different Ni-doped MIL-88A/GO weight ratios

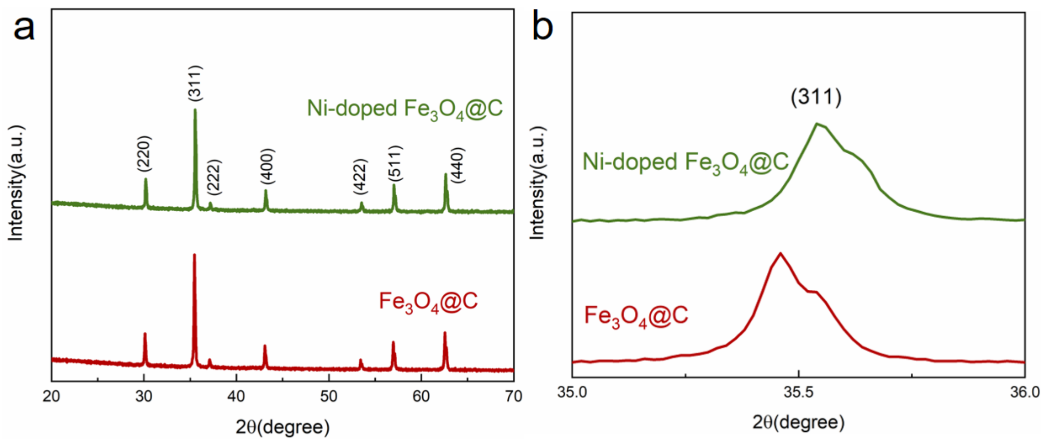

Fig. S7 XRD patterns of MIL-88A and Ni-doped MIL-88A derived Fe_3O_4@C and Ni-doped Fe_3O_4@C

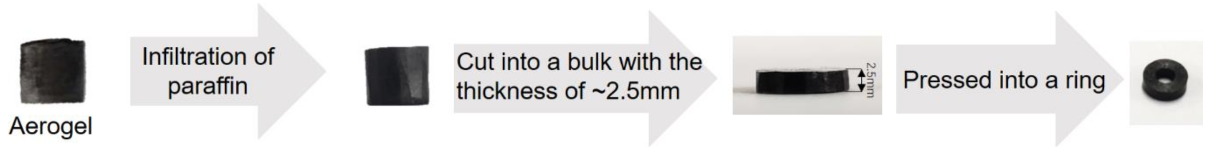

Fig. S8 The praperation process of concentric ring for the measurment of electromagnetic parameters

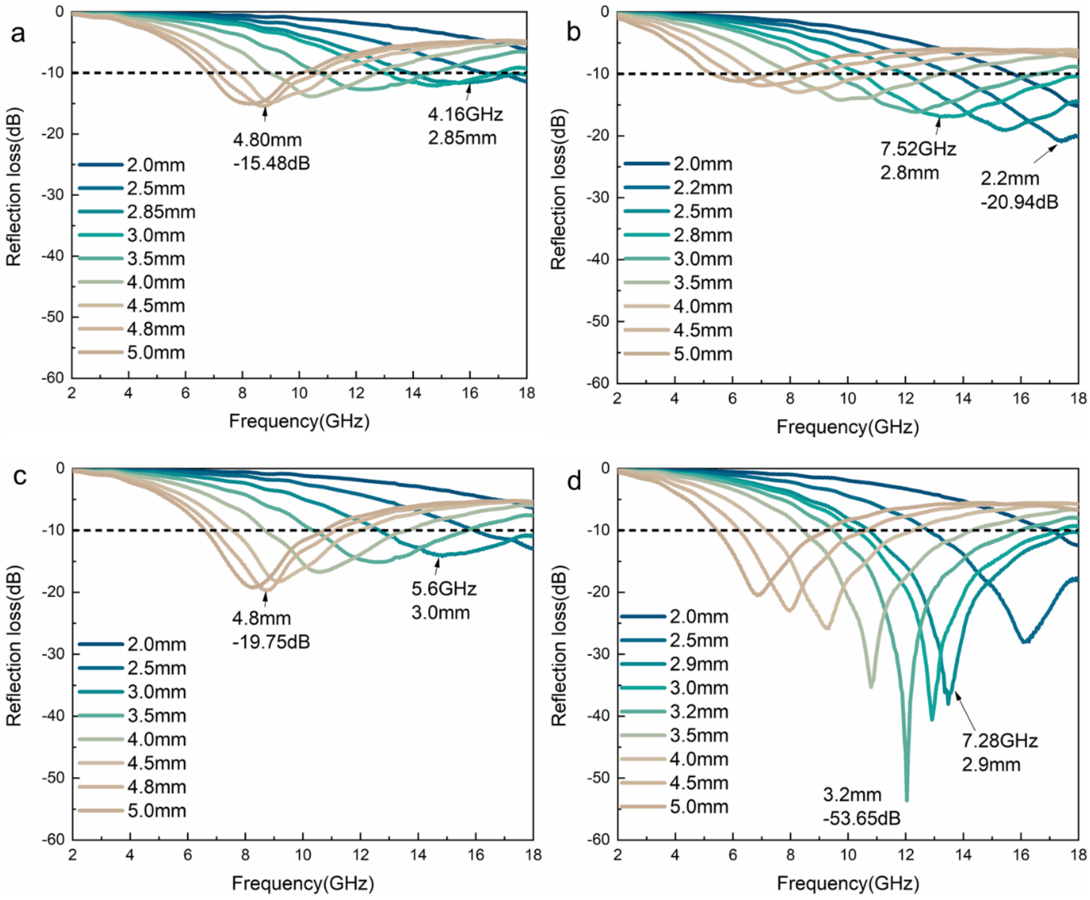

Fig. S9 RL−f curves of Fe_3O_4@C/rGO with the weight ratio of MOFs/GO at **a** 1: 2 **b** 3:2. RL−f curves of Fe_3O_4@C/rGO with the weight ratio of MOFs/GO at 1:1 treated at **c** 700°C and **d** 900°C

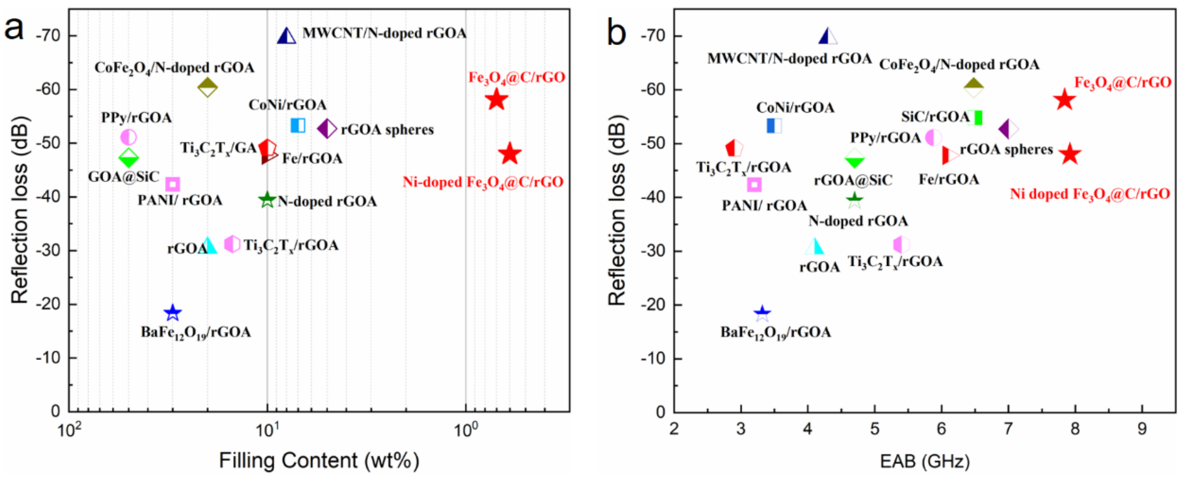

Fig. S10 a, b Comparison of the MA performance in view of the RL_{min}, EAB and filling content with reported rGO-based aerogels

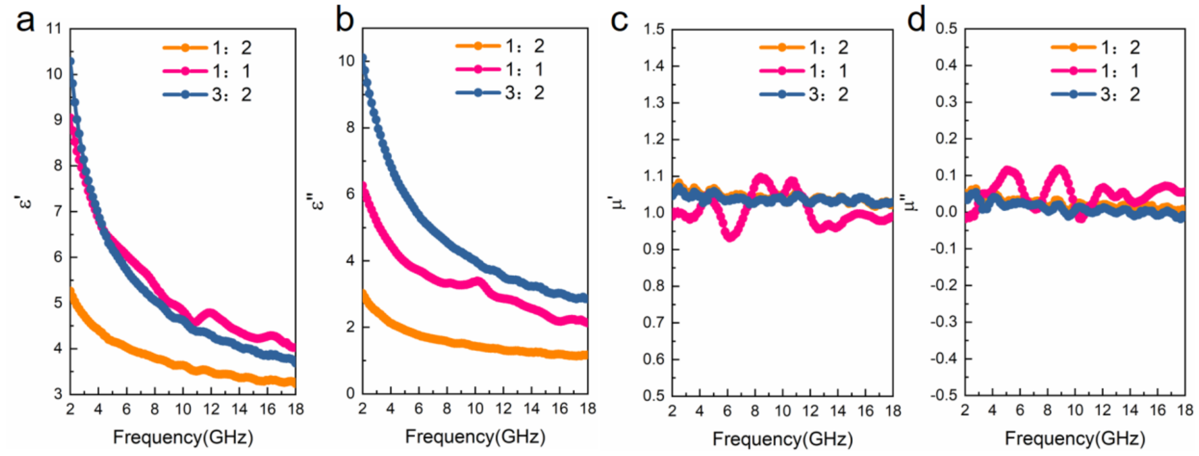

Fig. S11 a Real part and **b** imaginary part of complex permittivity, **c** real part and **d** imaginary part of the complex permeability of Fe_3O_4@C/rGO aerogels with the different weight ratios of MOFs and GO

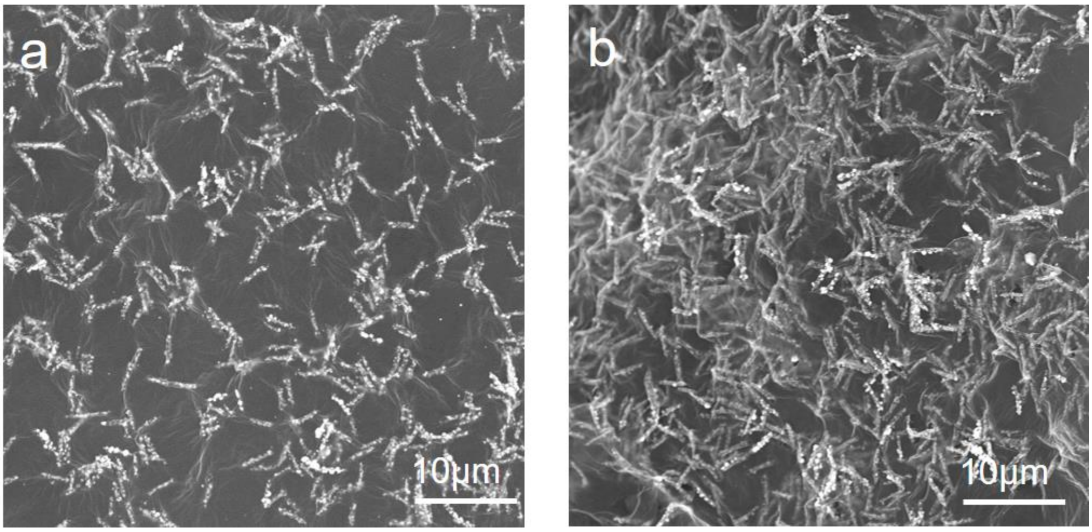

Fig. S12 SEM image of Fe_3O_4@C/rGO aerogels with the weight ratio of MOFs/GO at **a** 1: 1 **b** 3:2

Table S1 Comparison of the MA absorption performance of the previously reported MFe_2O_4 (M=Fe, Ni) composites

MFe_2O_4 (M=Fe, Ni) composites	Sample	RL_{min} (dB)	EAB (GHz)	Fill loading (wt%)	Refs.
Fe_3O_4 composites	Fe_3O_4/CMs composites	-60.3	6.4	5	[S1]
	Fe_3O_4/CNTs composites	-58.6	4.5	70	[S2]
	Fe_3O_4@C nanorings	-61.5	4.3	25	[S3]
	Fe_3O_4@TiO_2 composites	-23.3	6.0	16.7	[S4]
	ZnO/Fe_3O_4 composite	-36.2	4.02	60	[S5]
	Fe_3O_4/ZnO nanorods	-30.0	5.0	50	[S6]
	Fe_3O_4@PPy composites	-35.7	4.0	30	[S7]
	Fe_3O_4@PEDOT microspheres	-55.0	4.34	50	[S8]
	SiC/Fe_3O_4 nanocomposites	-51.0	4.9	50	[S9]
$NiFe_2O_4$ composites	$NiFe_2O_4$/r-GO composites	-42.0	5.3	50	[S10]
	$NiFe_2O_4$/carbon fibers	-55.8	4.0	25	[S11]
	Biomass porous carbon@$NiFe_2O_4$	-50.8	4.9	30	[S12]
	$NiFe_2O_4$@MnO_2 microspheres	-26.7	6.2	30	[S13]
	$NiFe_2O_4$-$Ti_3C_2T_x$-20 MXene	-24.7	7.68	25	[S14]
Fe_3O_4@C/rGO aerogel		-58.1	6.48	0.7	This work
Ni-doped Fe_3O_4@C/rGO aerogel		-46.2	7.92	0.6	

Table S2 Comparison of the MA absorption performance of the previously reported rGO-based aerogels

Sample	RL_{min} (dB)	EAB (GHz)	Fill loading (wt%)	Refs.
MWCNT/N-doped rGOA	-69.6	4.3	8	[S15]
CoNi/rGOA	-53.3	3.5	7	[S16]
$Ti_3C_2T_x$/rGOA	-49.1	2.9	10	[S17]
PANI/rGOA	-42.3	3.2	30	[S18]
$BaFe_{12}O_{19}$/rGOA	-18.35	3.32	30	[S19]
$CoFe_2O_4$/N-doped rGOA	-60.4	6.48	20	[S20]
SiC/rGOA	-54.8	6.5		[S21]
rGOA/SiC	-47.3	4.7	50	[S22]
PPy/rGOA	-51.12	5.88	50	[S23]
N-doped rGOA	-39.39	4.7	10	[S24]
rGOA	-30.53	4.1	20	[S25]
$Ti_3C_2T_x$/rGOA	-31.2	5.4	15	[S26]
rGOA spheres	-52.7	7	5	[S27]
Fe/rGOA	-47.8	6.1	10	[S28]
Fe_3O_4@C/rGO	-58.1	6.48	0.7	This work
Ni-doped Fe_3O_4@C/rGO	-46.2	7.92	0.6	

Table S3 Comparison of microwave absorption properties of reported high-performance MA absorbers

RL_{min} (dB)	EAB (GHz)	Thickness (mm)	Filling loading (wt %)	TLSW values	Ref
-58.1	6.48	2.5	0.007	21580	This work
-46.2	7.92	2.8	0.006	21780	This work
-75.2	5.4	2.15	0.0064	29512	ACSnano2021[S29]
-59.85	4	1.5	0.0824	1937	AFM2019[S30]
-41.5	2.7	1.4	0.07	1143	AS2021[S31]
-58.2	8.1	2.1	0.25	898	AM2016[S32]
-71.5	4.5	2.95	0.15	727	NML2021[S33]
-45.02	4.02	1.5	0.3	402	Small2021[S34]

Supplementary References

[S1] N. Yang, Z. Luo, S. Chen, G. Wu, Y. Wang, Fe_3O_4 nanoparticle/N-doped carbon hierarchically hollow microspheres for broadband and high-performance microwave absorption at an ultralow filler loading. ACS Appl. Mater. Interfaces **12**(16), 18952-18963 (2020). https://doi.org/10.1021/acsami.0c04185

[S2] X. Zeng, G. Jiang, L. Zhu, C. Wang, M. Chen et al., Fe_3O_4 nanoflower-carbon nanotube composites for microwave shielding. ACS Appl. Nano Mater. **2**(9), 5475-5482 (2019). https://doi.org/10.1021/acsanm.9b01076

[S3] X. Wang, F. Pan, Z. Xiang, Q. Zeng, K. Pei et al., Magnetic vortex core-shell Fe_3O_4@C nanorings with enhanced microwave absorption performance. Carbon **157**, 130-139 (2020). https://doi.org/10.1016/j.carbon.2019.10.030

[S4] J. Liu, R. Che, H. Chen, F. Zhang, F. Xia et al., Microwave absorption enhancement of multifunctional composite microspheres with spinel Fe_3O_4 cores and anatase TiO_2 shells. Small **8**(8), 1214-1221 (2012). https://doi.org/10.1002/smll.201102245

[S5] W. Ma, R. Yang, T. Wang, ZnO nanorod-based microflowers decorated with Fe_3O_4 nanoparticles for electromagnetic wave absorption. ACS Appl. Nano Mater. **3**(8), 8319-8327 (2020). https://doi.org/10.1021/acsanm.0c01728

[S6] Y. Chen, F. Zhang, G. Zhao, X. Fang, H. Jin et al., Synthesis, multi-nonlinear dielectric resonance, and excellent electromagnetic absorption characteristics of Fe_3O_4/ZnO core/shell nanorods. J. Phys. Chem. C **114**(20), 9239-9244 (2010). https://doi.org/10.1021/jp912178q

[S7] J. Guo, H. Song, H. Liu, C. Luo, Y. Ren et al., Polypyrrole-interface-functionalized nano-magnetite epoxy nanocomposites as electromagnetic wave absorbers with enhanced flame retardancy. J. Mater. Chem. C **5**(22), 5334-5344 (2017). https://doi.org/10.1039/c7tc01502j

[S8] L. Yan, X. Wang, S. Zhao, Y. Li, Z. Gao et al., Highly efficient microwave absorption of magnetic nanospindle–conductive polymer hybrids by molecular layer deposition. ACS Appl. Mater. Interfaces **9**(12), 11116-11125 (2017). https://doi.org/10.1021/acsami.6b16864

[S9] C. Liang, C. Liu, H. Wang, L. Wu, Z. Jiang et al., SiC–Fe_3O_4 dielectric–magnetic hybrid nanowires: controllable fabrication, characterization and electromagnetic wave absorption. J. Mater. Chem. A **2**(39), 16397-16402 (2014). https://doi.org/10.1039/c4ta02907k

[S10] J. He, X. Wang, Y. Zhang, M. Cao, Small magnetic nanoparticles decorating reduced

graphene oxides to tune the electromagnetic attenuation capacity. J. Mater. Chem. C **4**(29), 7130-7140 (2016). https://doi.org/10.1039/C6TC02020H

[S11] W. Li, H. Qi, F. Guo, X. Niu, Y. Du et al., $NiFe_2O_4$ nanoparticles supported on cotton-based carbon fibers and their application as a novel broadband microwave absorbent. RSC Adv. **9**(51), 29959-29966 (2019). https://doi.org/10.1039/c9ra05844c

[S12] Y. Wang, X. Gao, H. Zhou, X. Wu, W. Zhang et al., Fabrication of biomass-derived carbon decorated with $NiFe_2O_4$ particles for broadband and strong microwave absorption. Powder Technol. **345**, 370-378 (2019). https://doi.org/10.1016/j.powtec.2019.01.025

[S13] Y. Wang, Y. Fu, X. Wu, W. Zhang, Q. Wang et al., Synthesis of hierarchical core-shell $NiFe_2O_4$@ MnO_2 composite microspheres decorated graphene nanosheet for enhanced microwave absorption performance. Ceram. Int. **43**(14), 11367-11375 (2017). https://doi.org/10.1016/j.ceramint.2017.05.344

[S14] D. Shan, J. He, L. Deng, S. Yan, H. Luo et al., The underlying mechanisms of enhanced microwave absorption performance for the $NiFe_2O_4$-decorated $Ti_3C_2T_x$ MXene. Results Phys. **15**, 102750 (2019). https://doi.org/10.1016/j.rinp.2019.102750

[S15] R. Shu, Z. Wan, J. Zhang, Y. Wu, Y. Liu et al., Facile design of three-dimensional nitrogen-doped reduced graphene oxide/multi-walled carbon nanotube composite foams as lightweight and highly efficient microwave absorbers. ACS Appl. Mater. Interfaces **12**(4), 4689-4698 (2020). https://doi.org/10.1021/acsami.9b16134

[S16] H. Zhao, J. Cheng, J. Zhu, Y. Wang, Ultralight CoNi/rGO aerogels toward excellent microwave absorption at ultrathin thickness. J. Mater. Chem. C **7**(2), 441-448 (2019). https://doi.org/10.1039/c8tc05239e

[S17] Y. Li, F. Meng, Y. Mei, H. Wang, Y. Guo et al., Electrospun generation of $Ti_3C_2T_x$ MXene@graphene oxide hybrid aerogel microspheres for tunable high-performance microwave absorption. Chem. Eng. J. **391**, 123512 (2020). https://doi.org/10.1016/j.cej.2019.123512

[S18] Y. Wang, X. Gao, Y. Fu, X. Wu, Q. Wang et al., Enhanced microwave absorption performances of polyaniline/graphene aerogel by covalent bonding. Compos. Part B Eng. **169**, 221-228 (2019). https://doi.org/10.1016/j.compositesb.2019.04.008

[S19] T. Zhao, X. Ji, W. Jin, C. Xiong, W. Ma et al., Synthesis and electromagnetic wave absorption property of amorphous carbon nanotube networks on a 3D graphene aerogel/$BaFe_{12}O_{19}$ nanocomposite. J. Alloys Compd. **708**, 115-122 (2017). https://doi.org/10.1016/j.jallcom.2017.03.001

[S20] X. Wang, Y. Lu, T. Zhu, S. Chang, W. Wang, $CoFe_2O_4$/N-doped reduced graphene oxide aerogels for high-performance microwave absorption. Chem. Eng. J. **388**, 124317 (2020). https://doi.org/10.1016/j.cej.2020.124317

[S21] Y. Cheng, M. Tan, P. Hu, X. Zhang, B. Sun et al., Strong and thermostable SiC nanowires/graphene aerogel with enhanced hydrophobicity and electromagnetic wave absorption property. Appl. Surf. Sci. **448**, 138-144 (2018). https://doi.org/10.1016/j.apsusc.2018.04.132

[S22] Y. Jiang, Y. Chen, Y.J. Liu, G.X. Sui, Lightweight spongy bone-like graphene@SiC aerogel composites for high-performance microwave absorption. Chem. Eng. J. **337**, 522-531 (2018). https://doi.org/10.1016/j.cej.2017.12.131

[S23] B. Liu, J. Li, L. Wang, J. Ren, Y. Xu, Ultralight graphene aerogel enhanced with

transformed micro-structure led by polypyrrole nano-rods and its improved microwave absorption properties. Compos. Part A Appl. Sci. Manuf. **97**, 141-150 (2017). https://doi.org/10.1016/j.compositesa.2017.03.001

[S24] J. Xu, X. Zhang, H. Yuan, S. Zhang, C. Zhu et al., N-doped reduced graphene oxide aerogels containing pod-like N-doped carbon nanotubes and FeNi nanoparticles for electromagnetic wave absorption. Carbon **159**, 357-365 (2020). https://doi.org/10.1016/j.carbon.2019.12.020

[S25] C. Wang, Y. Ding, Y. Yuan, X. He, S. Wu et al., Graphene aerogel composites derived from recycled cigarette filters for electromagnetic wave absorption. J. Mater. Chem. C **3**(45), 11893-11901 (2015). https://doi.org/10.1039/c5tc03127c

[S26] L. Wang, H. Liu, X. Lv, G. Cui, G. Gu, Facile synthesis 3D porous MXene $Ti_3C_2T_x$ @RGO composite aerogel with excellent dielectric loss and electromagnetic wave absorption. J. Alloys Compd. **828**, 154251 (2020). https://doi.org/10.1016/j.jallcom.2020.154251

[S27] T. Li, D. Zhi, Y. Chen, B. Li, Z. Zhou et al., Multiaxial electrospun generation of hollow graphene aerogel spheres for broadband high-performance microwave absorption. Nano Res. **13**(2), 477-484 (2020). https://doi.org/10.1007/s12274-020-2632-0

[S28] H. Wang, H. Ma, Highly enhanced electromagnetic wave absorption bandwidth based on reduced graphene oxide-Fe aerogel composites. Nanotechnology **31**(9), 095711 (2020). https://doi.org/10.1088/1361-6528/ab59b4

[S29] L. Liang, Q. Li, X. Yan, Y. Feng, Y. Wang et al., Multifunctional magnetic $Ti_3C_2T_x$ MXene/graphene aerogel with superior electromagnetic wave absorption performance. ACS Nano **15**(4), 6622-6632 (2021). https://doi.org/10.1021/acsnano.0c09982

[S30] Y. Li, X. Liu, X. Nie, W. Yang, Y. Wang et al., Multifunctional organic–inorganic hybrid aerogel for self-cleaning, heat-insulating, and highly efficient microwave absorbing material. Adv. Funct. Mater. **29**(10), 1807624 (2019). https://doi.org/10.1002/adfm.201807624

[S31] X. Xu, S. Shi, Y. Tang, G. Wang, M. Zhou et al., Growth of NiAl-layered double hydroxide on graphene toward excellent anticorrosive microwave absorption application. Adv. Sci. **8**(5), 2002658 (2021). https://doi.org/10.1002/advs.202002658

[S32] Q. Liu, Q. Cao, H. Bi, C. Liang, K. Yuan et al., CoNi@SiO_2@TiO_2 and CoNi@air@TiO_2 microspheres with strong wideband microwave absorption. Adv. Mater. **28**(3), 486-490 (2016). https://doi.org/10.1002/adma.201503149

[S33] Y. Zhao, X. Zuo, Y. Guo, H. Huang, H. Zhang et al., Structural engineering of hierarchical aerogels comprised of multi-dimensional gradient carbon nanoarchitectures for highly efficient microwave absorption. Nano-Micro Lett. **13**, 144 (2021). https://doi.org/10.1007/s40820-021-00667-7

[S34] J. Xu, X. Zhang, Z. Zhao, H. Hu, B. Li et al., Lightweight, fire-retardant, and anti-compressed honeycombed-like carbon aerogels for thermal management and high-efficiency electromagnetic absorbing properties. Small **17**(33), 2102032 (2021). https://doi.org/10.1002/smll.202102032
